# Frequent Occurrence of Highly Expanded but Unrelated B-Cell Clones in Patients with Multiple Myeloma

**DOI:** 10.1371/journal.pone.0064927

**Published:** 2013-05-28

**Authors:** Jitra Kriangkum, Sarah N. Motz, Carina S. Debes Marun, Sandrine T. Lafarge, Spencer B. Gibson, Christopher P. Venner, James B. Johnston, Andrew R. Belch, Linda M. Pilarski

**Affiliations:** 1 Department of Oncology, Cross Cancer Institute, University of Alberta, Edmonton, Canada; 2 Department of Immunology, University of Manitoba, Winnipeg, Canada; 3 Manitoba Institute of Cell Biology, Winnipeg, Canada; University of North Carolina at Chapel Hill, United States of America

## Abstract

Clonal diversity in multiple myeloma (MM) includes both MM-related and MM-unrelated clonal expansions which are subject to dominance exerted by the MM clone. Here we show evidence for the existence of minor but highly expanded unrelated B-cell clones in patients with MM defined by their complementary determining region 3 (CDR3) peak. We further characterize these clones over the disease and subsequent treatment. Second clones were identified by their specific IgH-VDJ sequences that are distinct from those of dominant MM clones. Clonal frequencies were determined through semi-quantitative PCR, quantitative PCR and single-cell polymerase chain reaction of the clone-specific sequence. In 13/74 MM patients, more than one dominant CDR3 peak was identified with 12 patients (16%) being truly biclonal. Second clones had different frequencies, were found in different locations and were found in different cell types from the dominant MM clone. Where analysis was possible, they were shown to have chromosomal characteristic distinct from those of the MM clone. The frequency of the second clone also changed over the course of the disease and often persisted despite treatment. Molecularly-defined second clones are infrequent in monoclonal gammopathy of undetermined significance (MGUS, 1/43 individuals or 2%), suggesting that they may arise at relatively late stages of myelomagenesis. In further support of our findings, biclonal gammopathy and concomitant MM and CLL (chronic lymphocytic leukemia) were confirmed to originate from two unrelated clones. Our data supports the idea that the clone giving rise to symptomatic myeloma exerts clonal dominance to prevent expansion of other clones. MM and second clones may arise from an underlying niche permissive of clonal expansion. The clinical significance of these highly expanded but unrelated clones remains to be confirmed. Overall, our findings add new dimensions to evaluating related and unrelated clonal expansions in MM and the impact of disease evolution and treatment on clonal diversity.

## Introduction

Multiple myeloma (MM) is a hematological disorder involving malignant B-lineage cells. The need for therapy reflects the development of a clonal plasma cell population giving rise to symptomatic disease on the plasma cell dyscrasia (PCD) continuum; one that begins with monoclonal gammopathy of unknown significance (MGUS), a common entity found in 3% of individuals age 50 or older with about 1% progress to MM each year, followed by asymptomatic myeloma in the majority of cases prior to evolving into overt disease [1], [2]. Biologically, MM is comprised of cells primarily of post-switch isotypes with clonotypic immunoglobulin heavy chain (IgH) genes heavily mutated and lacking intraclonal heterogeneity [3]–[5]. MM also harbors complex genetic abnormalities with inherent genetic instability; a feature which is thought to be necessary for clonal evolution of the disease over time [6]. In recent years, novel treatments have improved patient outcome yet cure remains elusive [7]–[10]. The result is ongoing clonal evolution of the disease with an often changing clinical phenotype over time.

In general PCDs arise from the monoclonal expansion of a single transformed progenitor. We speculate that the dominant clone in MM may arise from a pool of cells that develop in a niche abnormally permissive for clonal expansion. The make-up of this clonal pool is poorly characterized. Questions remain regarding whether the cells are all derived from a common genetically related progenitor, a mixture of genetically distinct clones or a combination thereof. Eventual clonal dominance may suppress any subsequently arising clones [11]–[13].

Clinical evidence for the existence of two B-lineage clones in MM, whether related or unrelated, is unusual. Conventional means of identifying minor clones is limited to serum and urine protein electrophoresis. Using such techniques, biclonality is thought to be infrequent [3], [14], [15]. Because IgH undergoes class-switch recombination, multiple isotypes having the same VDJ rearrangement are detectable in MM [16]. Clonotypic μ transcripts are found in a majority of patients with IgG MM [16], [17]. In contrast, molecular analysis reported here reveals a considerably higher incidence of patients with apparent second clones. This has been shown in Waldenstrom's macroglobulinemia with two B-cell clones having distinct IgH-VDJ sequences identified in 3/19 patients despite detection of only one M-protein [18]. The incidence in of this phenomenon in MM or MGUS is unknown.

Here we describe the development of second clones arising in patients with MM, defined by the presence of productive IgH-VDJ rearrangements whose sequence is unrelated to that of the clinically-dominant MM clone. This was done through analysis of CDR3, clonotypic VDJ sequencing and clonal frequency analysis. We examined clonal changes over time, the extent to which dominance by the MM clone has been overcome and the extent to which new clones have expanded. We further confirmed our findings by single-cell PCR analysis of sorted B and plasma cells (PC) from peripheral blood (PB) and bone marrow (BM). Although all were present at relatively high frequency in PB and/or BM, most were clinically cryptic. This in-depth molecular analysis reveals a high incidence of highly expanded second clones in MM but not in MGUS. Second clones persisted over time and some harbored chromosomal abnormalities. Characterization of second clones suggests that they share at least some properties with tumor cells.

## Patients, Materials and Methods

### Ethics Statement

The study was approved by the University of Alberta, Alberta Health Services Institutional Review Boards and the Research Ethics Board at the University of Manitoba. Written informed consent was provided in accordance with the Declaration of Helsinki.

### Patient samples

BM aspirates and PB samples were from 43 MGUS and 74 MM patients randomly selected from the clinic at the Cross Cancer Institute, identified by consensus criteria. Samples were collected at diagnosis. Sequential samples were available from four patients (PT3, PT5, PT6 and PT8) to analyse clonal changes over time. Analysis was performed on purified mononuclear cells (MNC) from BM and PB. A patient with concomitant MM and CLL from Cancercare Manitoba provided purified CLL and MM cells.

### Polymerase chain reaction (PCR)

All PCRs were performed using T*aq* DNA polymerase (Invitrogen). Unless otherwise stated, amplification was run for 30 cycles at 94°C for 30 seconds, 60°C for 30 seconds, and 72°C for 30 seconds followed by extension at 72°C for 7 minutes. Primer sequences and combinations of primer sets for two-stage PCR are summarized in Supplementary Tables S1 and S2.

### Analysis of CDR3 and identification of clonotypic VDJ sequences

CDR3 regions were amplified from cDNA (RT-PCR) or genomic DNA (g-PCR) using FAM (carboxyfluorescein)-labeled FR3 and J_H_c or C_H_1 primers. These are consensus primers. DNA fragment analysis was performed on an ABI Prism 3130*xl* Genetic Analyzer (Applied Biosystems) and data were analysed by GeneMapper software version 4.0. Calculation of CDR3 followed Kriangkum, et al [18]. This method does not detect additional isotypes of the MM clone unless they are present at relatively high frequency and can be detected by consensus primers; this did not occur. Clonotypic VDJ was amplified by RT-PCR according to Taylor, et al [19]. Characterization of clonotypic VDJ is validated when its CDR3 sequence matches with that of DNA fragment analysis. Primers for clonal analysis were designed based on unique CDR1, CDR2 and CDR3 sequences.

### Clonal analysis by semi-quantitative PCR (semi-qPCR)

Genomic DNA (200 ng) was 5-fold serially diluted and used as template for clone-specific CDR1/CDR3 or CDR2/CDR3 primed amplification. The PCR was run for 35 cycles and products were analysed on 2% agarose gel electrophoresis. For nested PCR, 75 ng genomic DNA was 5-fold serially diluted and amplified by FR1c/JHc primers for 35 cycles. One microliter of first PCR reaction mix was amplified by clone-specific primers in the second PCR for additional 35 cycles and analysed by 2% agarose gel electrophoresis.

### Clonal analysis by quantitative PCR (qPCR)

qPCR quantifies the number of clonal cells by measuring the number of genomic copies of a given rearranged IgH-VDJ [20]. The analysis was performed with SYBR green using 2X SensiMix DNA kit (Quantace) and a DNA Engine Opticon 2 real-time PCR detector with the Opticon Monitor 3.1 software package (Bio-Rad). Two separate experiments were carried out: clone-specific CDR1/CDR3 or CDR2/CDR3, and β_2_m (internal control). Standards were plasmids containing each clonal specific VDJ or β_2_m DNA fragment; 10-fold serial dilutions were used along with test samples. Melting curves verified the specificity of the amplification.

### Single cell analysis

MNC were stained using fluorescein isothiocyanate-conjugated anti-CD20 (rituximab)[21] and phycoerythrin-conjugated anti-CD138. Isotype matched antibodies were negative controls. Using BD Influx™ cell sorter (Becton Dickinson), subpopulations of single cells were sorted. Small lymphocyte scatter gates were used when sorting for CD20^+^ cells and plasma cell scatter gates were used when sorting for CD138^+^ cells. The morphology of sorted cells was also confirmed in cytospins by microscopic examination. Individual cells were sorted into PCR tubes and processed as previously described [22]. Each single cell was analysed by nested PCR for clonal sequence and β_2_m. Clonal frequency was calculated as the percentage of cells positive to test reaction over the total number of cells positive to β_2_m.

### Fluorescence in situ hybridization (FISH) and immuno-FISH

Cytospin slides of MNC were stained with May-Grünwald-Giemsa or were immunostained as previously described [23], [24]. Sorting was as indicated above for single cell analysis. The immuno-FISH was evaluated from 100 stained cells. Seven probe sets were used: a commercial Vysis LSI D13S319 probe to detect deletion of 13q14 locus, LSI IgH dual color break apart probe to detect any translocation of the IgH locus, Vysis LSI IGH/CCND1-XT DF FISH probe set to detect t(11,14)(q13;q32), dual fusion LSI IgH/FGFR3 DF to detect t(4;14)(p16;q32), dual fusion LSI IgH/MAF DF to detect t(14;16)(q32;q23), Vysis CEP 1(D1Z5) was a control probe combined with a home-made probe targeting locus 1q21 to detect amplified 1q21, and a mixture of CEP 17 (D17Z1) and LSI TP53 to detect deletion of p53 locus [24]. Bright-field and fluorescence microscopy used the BioView Duet system to correlate the FISH staining (63×objective) pattern and immunostaining or morphology (40×objective).

### Selection of MM and CLL cells

MNC were stained using V450-conjugated anti-CD19, allophycocyanin-conjugated anti-CD5, phycoerythrin-conjugated anti-CD138 and 7-aminoactinomycin-D (7-AAD) according to the standard protocol. After excluding 7-AAD^+^ cells, CD19^+^CD5^+^ cells (CLL) and CD138^+^ cells (MM) were sorted for single cell analysis.

## Results

### In DNA fragment analysis, a dual CDR3 peak profile is frequent in patients with MM

**Figure 1:**
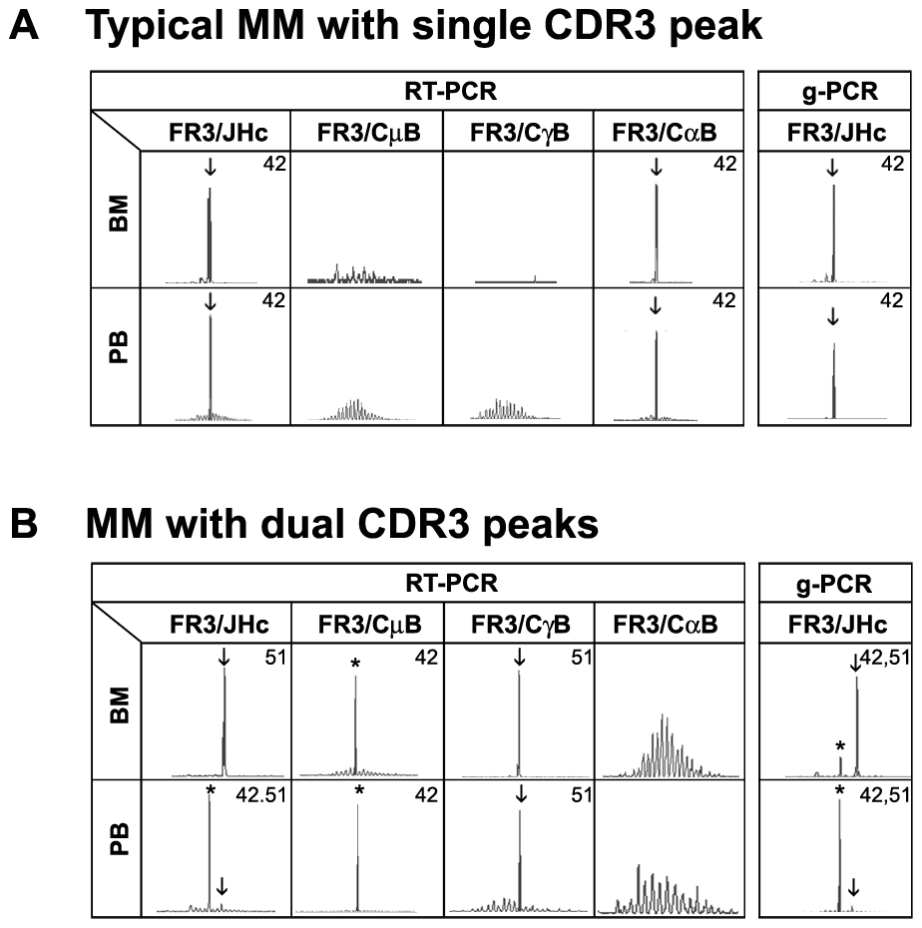
In DNA fragment analysis, a dual CDR3 peak profile is frequent in MM patients.

MM typically produces one monoclonal protein which is thought to reflect the single problematic clone giving rise to symptomatic disease. Traditionally this should be represented by a single peak profile in detecting CDR3 by DNA fragment analysis. Here, we show that this is not always true. CDR3s were profiled by RT-PCR and g-PCR using a universal Ig primer set, FR3/C_H_1 and/or FR3/J_H_c in 74 unselected MM patients. The expected single CDR3 peak profile was found in 61/74 patients. A representative result is shown in Figure 1A. Of the remaining 13 patients, 12 had two CDR3 peaks and 1 patient (PT9) had three CDR3 peaks (described below). Conventional serum protein electrophoresis indicated that 2 patients had biclonal gammopathies (PT6 and PT7) and 11 patients had a single clinically detected M-protein. One patient, PT13, was later shown to be biallelic and was excluded from further clonal analysis (described below). Table 1 summarizes the clinical data of 12 MM patients included in this study.

**Table 1:** Summary of pertinent clinical and laboratary data of MM patients with molecularly defined second clones.

ID^a^	Pertinent clinical and laboratory data	FISH analysis of BM PC
PT1	IgGλ; M protein, 9 g/L with minimal lambda light chain proteinuria and renal impairment	trisomy 13
PT2	IgGκ; M protein, 25 g/L	N/A
PT3	IgGκ; M protein, 96 g/L; CBC showed lymphocytosis at 7.6×10^9^/L. Approximately 49% of overall gated lymphoid cells were CD-19^+^ B-cells. There were also CD19^−^CD45^−^CD138^+^ cells with cytoplasmic kappa light chain restriction that accounted for 11% of overall ungated cells. Pathology report of BM biopsy identified 90% myeloma cells and a slight increase of CD20-positive lymphoid cells is noted, suggesting a minimal involvement of the low-grade B-cell lymphoma seen in the blood.	del(13q); t(11;14)
PT4	IgGλ; M protein 17 g/L	N/A
PT5	IgDλ; M protein, 6.3 g/L with free lambda light chain; responded to treatment during this study, followed by a relapse	t(11;14), trisomy 1, 4, 11, 14 and 17
PT6	Biclonal gammopathy; IgGλ and IgGκ; M protein, 16 g/L; successfully treated during this study, subsequently relapsed	BM aspirates from two different anatomical sites yielded distinct genetic abnormalities: sternum BM, del(13q) and amp(1q21); iliac BM, t(11;14)
PT7	Biclonal gammopathy, IgGκ and IgAκ; M protein, 56 g/L	del(13q); t(11;14); trisomy 11
PT8	IgGκ; M protein, 26 g/L; responded well to thalidomide for five years, eventually relapsed and resisted every treatment options	N/A
PT9	IgGκ; M protein 55 g/L; previously hypothyroidism, peptic ulcer disease, hypertension and coronary artery disease	del(13q); t(14;16), del p53
PT10	IgGκ; M protein 60 g/L,	translocation 14q32; trisomy 1
PT11	IgAκ; M protein 28 g/L; successfully treated with Velcade/Cyclophosphamide/Prednisone	N/A
PT12	IgAκ; M protein 37 g/L	trisomy 1

a Samples for molecular analysis were collected at diagnosis. For PT3, PT5, PT6 and PT8, multiple samples were studied.

N/A, not applicable.

A representative CDR3 profile with two dominant peaks is shown in Figure 1B where an IgG MM patient (PT1) expressed an MM γ peak (FR3/CγB, CDR3 = 51 nt) and a second non-MM μ peak (FR3/CμB, CDR3 = 42 nt). The cellular distribution measured by g-PCR where each cell contributes only a single copy of rearranged VDJ template showed that the IgH-VDJ of the MM peak was dominant in BM, while the IgH-VDJ corresponding to second CDR3 peak was dominant in PB. This indicates that the two IgH-VDJ rearrangements were present in cell populations from different anatomical locations.

In contrast to MM, the occurrence of two CDR3 peaks was detected in only 1/43 (2%) patients from an age-matched MGUS cohort of patients. Second clones appear to become detectable at stages much later than that of MGUS. However, this does not necessarily imply that they are not present but rather that they may not be readily identified using present technology. The single MGUS patient with two IgH-VDJ rearrangements does not appear to be high risk and has not progressed to MM over 6 years of follow-up.

### Second CDR3 peaks are derived from productively rearranged IgH

The rearranged IgH-VDJ genes corresponding to dominant CDR3 peaks for PT1-12 were fully characterized. The clinically related MM and the second rearranged IgH-VDJ sequences were shown to be productively rearranged without stop codons and with proper deduced amino acid sequences as expected for V_H_ gene products. For PT9, a third CDR3 peak had a frameshift at the VDJ junctions; this sequence was not part of later clonal analysis as it was shown to be biallelic within the MM clone. Table 2 summarizes isotypes, VDJ gene usage, mutational status, and CDR3s of rearranged IgH corresponding to clinically-defined MM and presumptive second clones for PT1-12. These patients fall into three groups based on the isotype of the second peak: Group I: pre-switch isotypes (μ and δ) (five patients, PT1-5), Group II: a post-switched isotype (γ or α) (five patients, PT6-10) and Group III: combined pre-and post- switched isotypes (μ, γ and/or α) (two patients, PT11-12). In each patient, the second clonal VDJ rearrangement was different from that of the dominant MM clone. All clinically-defined MM IgH-VDJs were hypermutated (2.4–15.0%) with CDR3s of 30–54 nt. The second rearranged IgH genes were also hypermutated (3.1–13.5%) with CDR3s of 18–51 nt, except for two unmutated sequences with long CDR3s (57 and 78 nt). Intraclonal heterogeneity was observed in second rearranged IgH-VDJ for PT8, PT11 and PT12.

**Table 2:** Characteristics of the second rearranged IgH-VDJ compared to that of MM.

		Isotype^a^		IgH-VDJ - MM peak					IgH-VDJ - 2^nd^ peak				
Group	ID	MM peak	2^nd^ peak	V_H_	D_H_	J_H_	%V_H_ Mut	CDR3, nt	V_H_	D_H_	J_H_	%V_H_ Mut	CDR3, nt
I	PT1	γ	μδ	1–46	3–3	4	10.42	51	3–23	3–10	4	8.33	42
	PT2	γ	μδ	3–30	2–21	5	12.23	54	1–8	3–10	4	3.78	39
	PT3	γ	μδ	3–30	4–17	6	2.44	51	3–43	6–19	4	1.05^b^	57
	PT4	γ	μδ	3–21	2–21	3	8.07	36	1–18	2–15	5	3.82	30
	PT5	δ	μδ	1–58	4–23	4	15.03	45	1–69	3–3	6	0^b^	78
II	PT6^c^	γ	γ	4–30	3–3	4	10.21	63	1–18	2–15	4	9.72	42
	PT7^c^	γ	α	4–4	3–16	4	9.03	30	3–48	3–3	6	10.07	45
	PT8^d^	γ	α	3–23	3–3	4	5.90	51	3–21	1–20	4	9.71	45
	PT9^e^	γ	α	3–15	6–6	3	7.82	42	4–34	6–6	3	3.14	48
	PT10	γ	γ	3–23	2–21	5	9.03	54	4–4	6–19	4	9.03	33
III	PT11^d^	α	μγα	3–21	1–1	5	5.53	36	3–74	5–18	4	5.90	18
	PT12^d^	α	μα	3–30	6–13	5	3.14	45	3–11	2–21	4	13.54	51

a Isotypes of CDR3 peaks were initially identified by FR3/C_H_1 RT-PCR. They were subsequently confirmed by RT-PCR using a clonal specific CDR3 primer (sense) and a C_H_1 primer (antisense).

b unmutated, under 2% cut-off.

c Biclonal gammopathy was identified by serum protein for PT6 (IgG-λ and IgG-κ) and PT7 (IgG and IgA).

d Second rearranged IgH-VDJ exhibited intraclonal heterogeneity.

e PT9 had one clone with a productive and a non-productive rearrangement, and one clone with only a productive rearrangement. Rearranged IgH-VDJ of non-productive allele was not shown.

### Second rearranged IgH are derived from non-MM cells

There are two possibilities for the source of second productively rearranged IgH-VDJ. First, it may be a second productive IgH rearrangement within the MM cells (biallelic) or second, it may be present in cell populations independent from the dominant MM clone (biclonal). Allelic exclusion ensures that two productive rearrangements occur in the same cell only rarely, if at all. Biallelic rearrangements in each cell, if present, will always be distributed together to yield equal copy numbers of DNA template for both IgH-VDJ segments. Thus the corresponding peak heights in g-PCR, clonal frequencies in semi-qPCR and qPCR, and anatomic distributions should be equal. Conversely, if the two IgH-VDJ rearrangements are in different cells, the two independent clones are likely to have different frequencies, may be found in different cell types and may inhabit different anatomic locations.

**Figure 2:**
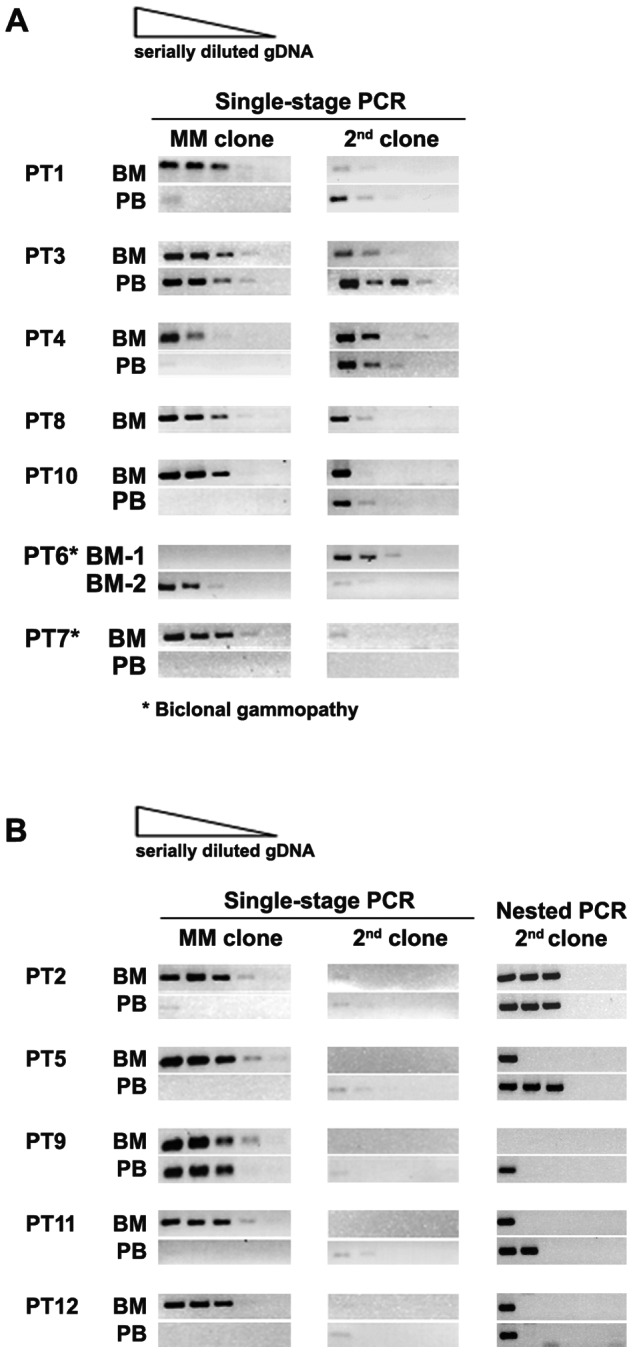
Clonal frequency of molecularly-defined second clone is different from that of clinically-defined MM clone. Distribution of clinically related MM and second clones in BM and PB were determined by semi-qPCR. For all samples tested, five-fold serially diluted gDNA aliquots (1/1, 1/5, 1/25, 1/125, 1/625) were amplified by clone-specific primers in single-stage PCR and analysed on agarose gel electrophoresis (A). Clonal abundance was estimated by comparing the highest dilution of gDNA that gave positive band. Samples were collected at diagnosis with a few exceptions: PT8, BM was collected at a relapse, referred to as BM-3 in Figure 5; PT6, BM-1 was collected from sternum, BM-2 was collected from ilium; PB of PT7 was collected after initial chemotherapy. Nested PCR of serially diluted gDNA aliquots was performed in patients whose second clones were less frequent and difficult to detect by single-stage PCR (B).

The relative frequencies of second rearranged IgH sequences were compared against those of the clinically-related MM sequence in each patient by semi-qPCR, qPCR and relative peak heights of CDR3s by g-PCR (Figure 2 and Table 3). We show that clonal abundance measured by semi-qPCR is consistent with qPCR and relative peak heights of CDR3 in DNA fragment analysis. Unequal frequencies between the two rearranged IgH-VDJs were observed throughout. This is indicative of two independent clones rather than a biallelic source of IgH-VDJs. For PT3, the frequencies of two rearrangements appear similar, but longitudinal analysis and chromosomal studies confirmed that they were distinct (described below).

**Table 3:** Clonal frequencies of the two rearranged IgH-VDJs identified in MM.

		Clonal frequencies measured as% of MNC for clinically-related/molecularly-defined second clone^c^		Relative peak height measured by g-PCR for clinically-related/molecularly-defined second clone	
ID^a^	%PC^b^	BM	PB	BM	PB
PT1	20	23/3.3	3.2/10	++++/+	+/++++
PT2	20	23/1.5	1/1.7	++++/++	++/+++
PT3	80–90	37/24	41/31	++++/++	++++/++
PT4	10	10/11	1/14	+++/++	++/++++
PT5	50	47/0.06	0.5/1.2	++++/+	+/++
PT6^d^	12	0.3/3.3	<0.01/<0.01	+/++++	±/±
PT7^d^	80–90	73/1	0.01/0.1	++++/+	+/+
PT8	20–30	35/3.3	N/A	++++/+	N/A
PT9	50–60	45/<0.01	23/0.2	++++/+	++++/+
PT10	23	30/2.5	<0.01/3.5	++++/++	±/++++
PT11	40	31/0.2	0.04/0.5	++++/+	+/+++
PT12	80–90	50/0.1	0.3/0.1	++++/++	+/++

a BM and PB samples were taken at presentation with a few exceptions: a. PT7 PB was taken after initial chemotherapy, b. PT6 referred to as BM-2 in Figure 3 and c. PT8 referred to as BM-3 in Figure 4, matching PB was not available. PT3 and PT5 referred to BM-1 and PB-1 in Figure 3. The percentages noted (both the clinically determined and molecularly defined) are listed as the percentage of total mononuclear cells.

b Based on pathology report on BM aspirate.

c Clonal frequencies were measured by qPCR. Cells were assumed to have only one copy of rearranged IgH-VDJ and two copies of β_2_m. Clonal frequency was calculated as the number of cells positive for IgH-VDJ over the number of cells positive for β_2_m×100.

d Biclonal gammopathy.

N/A, sample not available.

Single-cell studies of PT4 and PT10 further supports the hypothesis that clinically-defined MM and second rearranged IgH were from different cell populations. The latter was detected in CD20^+^138^−^ B-cells, while the MM clone was found exclusively in CD20^−^CD138^+^ cells (Figure 3A). None of the single cells carried two VDJ sequences, verifying the biclonal distribution of the two IgH-VDJs. The second clonal frequencies in PT4 and PT10 were shown to be 10% and 19% in BM B-cells and 25% and 10% of PB B-cells, respectively. In contrast, two productive rearrangements were found in all individual cells from PT13 indicating that they were biallelic (Figure 3B) and they were thus excluded from further clonal analysis. The results for PT13 confirm that our methods accurately identify biallelic rearrangements if present.

**Figure 3:**
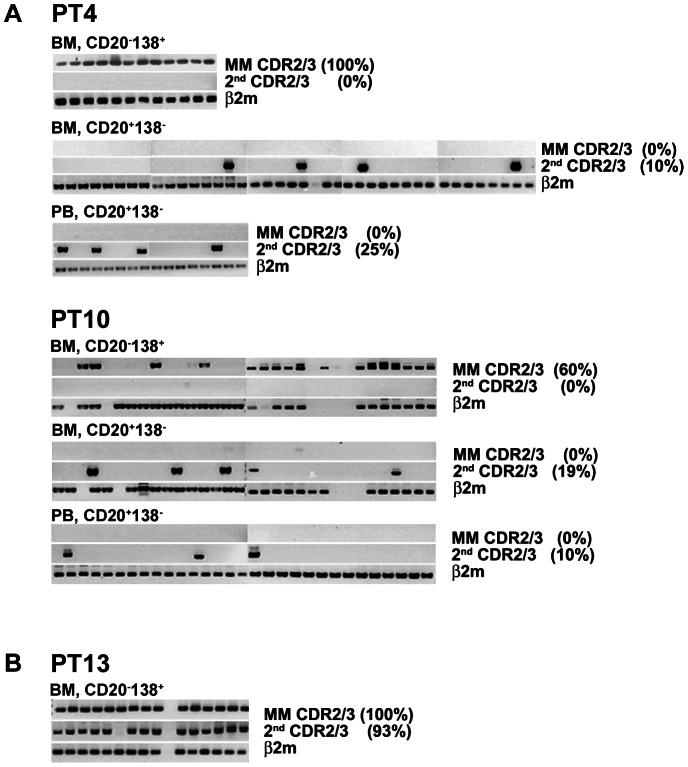
The clinically-defined MM clone and molecularly-defined second clone are derived from different cell populations. Single cells were sorted by phenotypic markers and clonality was determined by nested PCR using selected set of clone-specific CDR2/CDR3 or β_2_m primers as internal control. Each column shows PCR results obtained from one cell. Clonal frequency was calculated as the percentage of cells that were positive in test reaction over the total number of cells positive in internal control and is shown at the end of each row. For PT4 and PT10 (A), the MM clone includes most of CD138^+^ population, while the second clone is among CD20^+^138^−^ cells. PT4 BM and PB samples used in this analysis were taken 2 years after those analysed by qPCR (Table 2). For PT13 (B), MM cells carried two productively rearranged IgH; PT13 was not part of this study but is shown here to illustrate the pattern of IgH-VDJs when both are present in the same cell (biallelic).

In summary, MM patients with two IgH-VDJ rearrangements were confirmed to have two distinct clones: the dominant, clinically relevant MM clone and a molecularly-defined second clone. The two clones occur at widely different frequencies, are found in different anatomical locations (BM vs PB), are found in different cell types (PC vs B), and on single cell analysis are found only in individual cells that lack the alternate rearrangement.

### Frequencies of molecularly-defined second clones are higher than those of antigen-stimulated B-cells

Semi-qPCR and qPCR indicated that dominant-MM clones are most abundant in BM and their frequencies are different from those of molecularly-defined second clones (Figure 2 and Table 3). Second clones are present at frequencies ranging from <0.01–24% of BM MNC and from <0.01–31% of PB MNC. These frequencies are higher than those of circulating, antigen stimulated B-cells. In healthy donors, clonal frequencies of B-cells generated from HBV vaccination ranged 0.01–0.03% of B-cells, measured against polyvalent vaccine [25], [26]. This represents approximately 0.001% of PB MNC for antigen-expanded normal B-cells. The frequency of molecularly-defined second clones in BM, PB, or both is therefore ∼1–3 logs greater than that of normal antigen-stimulated B-cells. Overall, clinical monoclonal B lymphocytosis (MBL) was not detected by conventional means in these patients with overt MM. Furthermore, the cellular frequencies of most second clones in PB estimated by molecular techniques exceeded those reported for low-count MBL. In contrast, the incidence of molecularly-identified second clones in our age-matched MGUS cohort was low indicating few MBL-like clones in a population at high risk for developing MM

### Molecularly-defined second clones persist over time and throughout treatment

**Figure 4:**
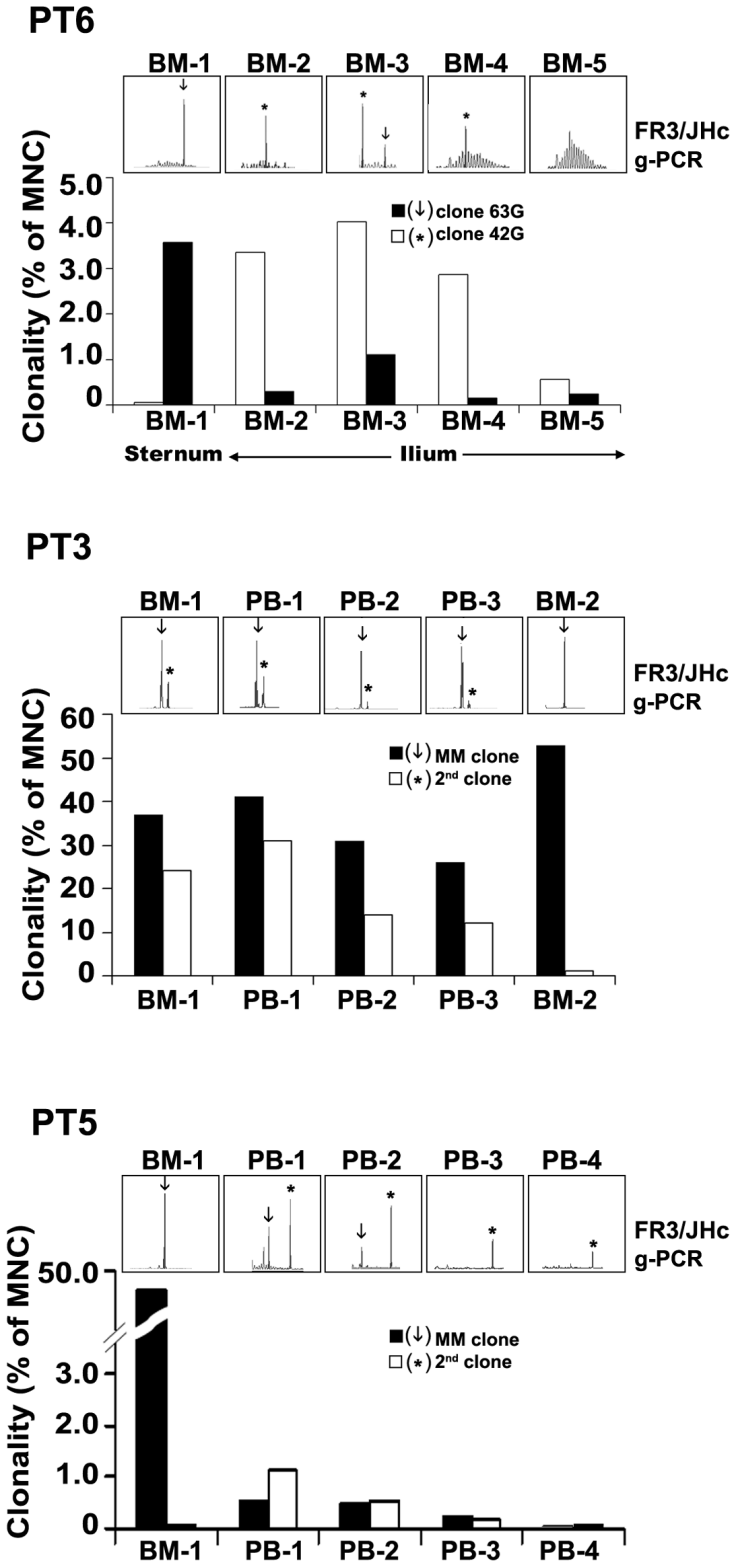
Molecularly-defined second clones persisted over time and treatment. A series of BM and PB specimens were analysed by g-PCR for CDR3 profiling and clonal frequencies were measured by qPCR as percentages of MNC. For PT6, BM-1 to -5 were taken at 0, 2, 9, 12, and 18 months respectively. For PT3, BM-1 and PB-1 were taken at diagnosis. PB-2, PB-3 and BM-2 were taken at 1, 5, and 24 months later. For PT5, BM-1 and PB-1 were taken at diagnosis. PB-2, PB-3 and PB-4 were taken at 2 weeks, 2 months, and 7 months later.

Three patients had multiple samples available for longitudinal analysis to evaluate the incidence of second clones throughout the course of disease (Figure 4).

First, in PT6 two distinct rearranged VDJ-Cγ gene segments were identified, consistent with the clinically detected elevated IgG-κ and IgG-λ in serum (data not shown). Five BM samples were collected over 18 months and both clonal frequencies were determined. CDR3 analysis clearly showed that clone 63G (CDR3 = 63 nt) predominated in sternal BM (BM-1), while clone 42G (CDR3 = 42 nt) was frequent in iliac BM (BM-2, Figures 2 and 4). The two IgH-VDJ rearrangements do not co-distribute. The differing frequencies in the two different BM locations for each IgH-VDJ rearrangement confirm that these represent two distinct clones and are thus biclonal not biallelic. Upon treatment, CDR3 spikes gradually declined and shifted toward a polyclonal profile (BM-5), in parallel with reduced partner clone frequencies shown by qPCR (Figure 4) and the dominant M-protein decreased from 16 to 1 g/L. For the set of BM samples analysed, isolated MNC contained 3–12% of PC by classic morphology. Thus, the% clonality shown by qPCR in Figure 4 was as expected if most of the PC were clonotypic cells.

Secondly, PT3 had a clinically related γ clone with CDR3 = 51 nt, yet RT-PCR for FR3/CμB and FR3/CδB revealed a second CDR3 peak at 57 nt (data not shown). Both clones had distinct IgH-VDJ sequences (Table 2). The clinically-defined MM clone accounted for 37% of MNC in BM-1, which two years later increased to 53% in BM-2. The molecularly-defined second clone comprised 24% of MNC in BM-1 but declined to 1% in BM-2, in agreement with the CDR3 profiling. For PT3, this large difference in clonal frequencies for BM-2 (53% for the MM clone and 1% for the second clone) confirms that they represent distinct and independently distributing clones. In blood (PB-1, PB-2, PB-3), over a five month period, the MM clone made up 26–41% of MNC and the second clone made up 12–31% of MNC. The presence of a second B-cell clone was also supported by clinical report of increased PB B-cells (Table 1). Both clones persisted during treatment and the single dominant M-protein in serum remained unchanged at about 90 g/L, indicating the lack of therapeutic response.

Thirdly, in PT5 a clonotypic VDJ-Cδ (CDR3 = 45 nt) and a second CDR3 peak identified by RT-PCR in blood samples as present both IgM and IgD transcripts (CDR3 = 78 nt) (data not shown). Both clones had distinct IgH-VDJ sequences (Table 2). Interestingly, this second clone carried unmutated VH1-69/DH3-3/JH6 gene segments similar to those found in CLL. The clinically-related MM clone predominated in BM (47% of MNC at diagnosis), while the second clone was more frequent in PB (1.2% of MNC in pre-treatment PB-1). The independent localization of the MM IgH-VDJ as compared to the second IgH-VDJ, in BM vs PB, confirms that they independently distribute and represent two distinct clones. Successful treatment was indicated by a significant decrease of the single M-protein from 6.3 to 1.7 g/L and the decline of clonal frequencies (PB-1-PB-4). Despite treatment, the second clone remained detectable by CDR3 profiling in PB-4.

Overall, longitudinal analysis showed that molecularly-defined second clones distributed independently from the MM clone, were present at differing frequencies, could be detected over time and resisted treatment. Their clinical significance is as yet unclear.

### Chromosomal characteristics of molecularly-defined second clones are distinct from those of the clinically-related MM clone

Chromosomal abnormalities were identified by FISH in aggregate PC from 8/8 patients analysed and the results are presented in Table 1. Complex chromosomal abnormalities were found in BM from 6/8 patients analysed with the panel of probe sets. Chromosomal abnormalities in second clones were studied by immuno-FISH, a method that combines isotype staining and FISH analysis. Conclusive results could be reached for PT3 and PT10 despite the probability that some non-clonal cells may be present in the isotype-selected population.

For PT3 who had both IgG restricted MM and an IgM second clone, del(13q) and t(11;14) were detected in total PC. The same abnormalities were detected in 54% and 61% of IgG^+^ cells (inclusive of MM PC) respectively. No abnormalities were detected in IgM^+^ cells which include the second clone. Thus the MM clone has cytogenetics distinct from those of the second clone.

For PT10, single cell analysis showed that second clone was among CD20^+^138^−^ cells, distinct from CD138^+^ MM cells (Figure 3). Immuno-FISH was performed in sorted BM PC (CD138^+^) and PB B-cells (CD20^+^138^−^). For CD138^+^ cells, translocation 14q32 was shown in 24% and trisomy 1 in 25% of IgG^+^ cells (the presumptive MM clone), in agreement with FISH data collected from aggregate PC. For CD20^+^138^-^ cells, IgG^+^ B-cells exhibited 16% trisomy 17 and 14% deletion p53. Thus, the MM clone and its partner second B-cell clone harbor distinct chromosomal abnormalities.

For PT6, partner clones had the same IgG isotype and predominated in different anatomical sites in BM (Figures 2 and 4). Distinct chromosomal abnormalities were identified by comparing FISH data from multiple BM samples (Table 4). In BM-1, a sternal biopsy where clone 63G was most frequent, del(13q) and amp(1q21) were shown in 91% and 99% of PC respectively; t(11;14) was not detected. In BM-2, a diagnostic iliac biopsy, where clone 42G was dominant, t(11;14) is present in 67% of PC. Since clone 63G did not exhibit t(11;14), we concluded that t(11;14) has come from 42G. Thus the two clones in PT6 have different anatomic localizations, different frequencies, and very different cytogenetics.

**Table 4:** FISH analysis of PT6 identifies two distinct sets of abnormalities.

	% of PC				
Chromosomal abnormalities	BM-1	BM-2	BM-3	BM-4	BM-5
del(13q)	91	15	7	3	5
amp(1q21)	99	4	0	0	0
t(11;14)	0	67	57	40	26

Overall, FISH and immuno-FISH analyses suggested that chromosomal abnormalities may be present in a second clone. Distinct chromosomal characteristics further support the IgH-VDJ analysis indicating that they are clonally unrelated.

### A second clone expands during the course of MM

**Figure 5:**
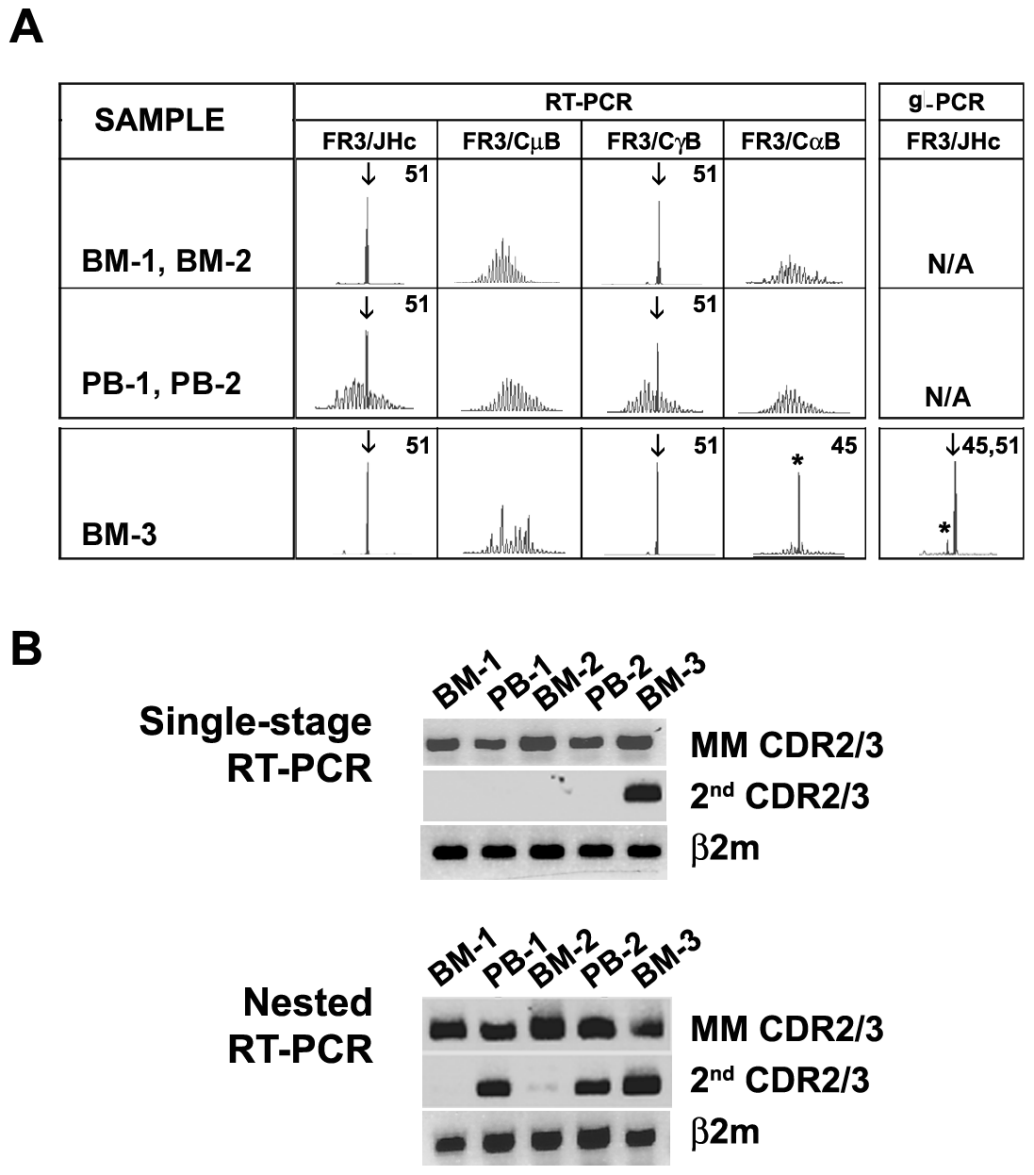
A second clonal expansion occurred during the course of MM. CDR3 profiling of patient PT8 is shown in (A). A similar profile was noted between BM-1 and BM-2 or PB-1 and PB-2, thus only one set of data is presented. ↓, clinically related MM peak; *, molecularly-defined second peak. CDR3 lengths in nt for these peaks are shown at top right of each panel. Clonal transcripts were determined by single-stage or nested RT-PCR (B). BM-1 and PB-1 were collected at diagnosis; BM-2 and PB-2 were collected a year later This patient received thalidomide treatment for six years. BM-3 was collected at a relapse seven years after diagnosis.

Clonal analysis in PT8 demonstrates the expansion of a second clone over the disease course. CDR3 profiling and clonal transcript were analysed in multiple PT8 samples collected at diagnosis (BM-1/PB-1), at follow-up one year later (BM-2/PB-2) and at relapse seven years after diagnosis (BM-3). In CDR3 analysis, the expected MM γ peak was found in all samples, but the second α peak was seen only in BM-3 (Figure 5A), after about 6 years of treatment with thalidomide. This suggests that its expansion occurred after the onset of MM. Clonal transcripts of both clones were detected by their respective unique CDR2/CDR3 sequences in RT-PCR (Figure 5B). Single-stage CDR2/CDR3 RT-PCR readily confirmed the presence of the second clone in relapse BM-3, but not in BM or PB samples taken at diagnosis or a year later. However, using nested RT-PCR, transcripts of the second clone became detectable in PB-1 and PB-2, but remained undetectable in BM-1 and BM-2. In summary, longitudinal analysis of PT8 suggests that precursor cells for the second clone existed in the repertoire for at least several years (identified in PB at diagnosis) before expanding to a frequency detectable by CDR3 analysis.

### Different clonal origins characterize concomitant MM and CLL

**Figure 6:**
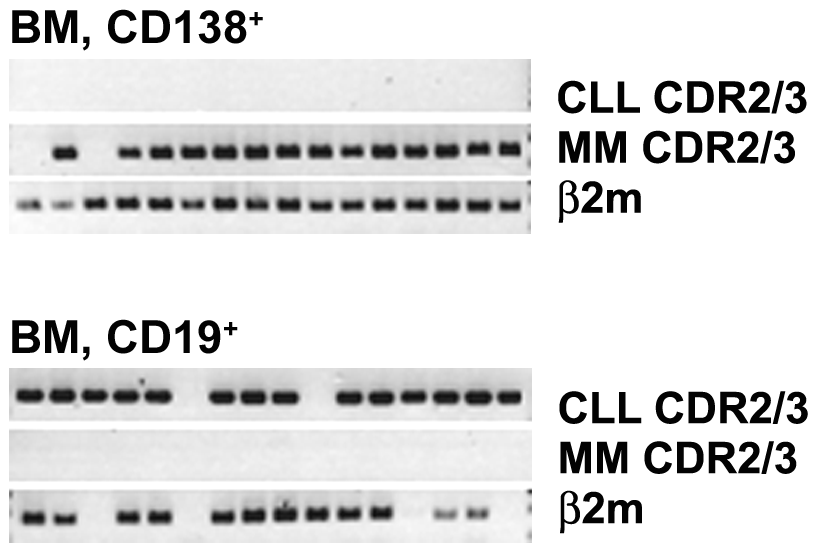
Biclonality characterizes a patient with concomitant MM/CLL (PT14). MM and CLL cells selected by phenotypic marker were sorted into single cells and clonality was analysed by nested-PCR using selected set of clone-specific CDR2/CDR3, or β_2_m primers as an internal control. Each column shows PCR results from one cell.

The frequent occurrence of second clones in MM led us to speculate that they could serve as a potential source of concurrent malignancies. In two patients with biclonal gammopathies (PT6-7), the transformation may have already occurred. As further proof of principle, we analysed BM from a patient with concomitant MM and CLL (PT14) to determine the clonal relationship between these two malignancies. Clonotypic VDJ sequences derived from sorted CLL cells (CD19^+^CD5^+^) and MM cells (CD138^+^) had distinct IgH-VDJ rearrangements. Clonotypic IgM derived from CLL cells utilized unmutated (0%) VH3-11/DH3-10/JH5 gene segments with CDR3 = 57 nt (CARDLVLYYGSGSYYNWFDPW); clonotypic IgG derived from MM cells utilized mutated (8.3%) VH1-18/DH6-19/JH5 gene segments with CDR3 = 42 nt (CARDAGGGSRYWFDPW). By single cell analysis, the signature sequence of the CLL clone was found only in CD19^+^CD5^+^ BM cells (B cells) and that of MM clone was found only in CD138^+^ BM cells (PC) (Figure 6). None of the single cells carried two VDJ sequences. The analysis here confirms that for PT14, concomitant MM and CLL are derived from two separate clones.

## Discussion

Although most MM patients display a single monoclonal protein produced by the dominant myeloma clone, we show here that a considerable number carry a highly expanded second clone. These second clones are usually B-cells that do not produce detectable M-protein and do not share a rearranged IgH-VDJ with the clinically-relevant MM clone. Interestingly they are present at a cellular frequency that considerably exceeds and therefore distinguishes them from antigen-stimulated B-cells or low-count MBL. Some second clones exhibit chromosomal abnormalities, persist over time and treatment and can remain “dormant” in the repertoire long before extensive clonal expansion occurs. The observation in this study that biclonal gammopathy and concomitant MM and CLL originated from two unrelated clones supports the idea that second clones may sometimes lead to second malignancies. This speculation gains weight from the observation that for one patient, the second clone expanded to high frequency only after years of treatment, including a long period on thalidomide, even though a very low frequency of such cells existed at diagnosis. This suggests that treatment may weaken dominance of the MM clone and/or alter the supporting microenvironmental niche, thereby occasionally allowing expansion to detectable levels of unrelated clones. The role that these other malignant clones may play in the development of the recently described “second primary malignancies” associated with long-term lenalidomide use is not clear. However, the possibility that they may be a potential source in this therapeutic context is compelling and warrants further study.

MM carrying two clones were shown in 12/74 patients (16%), two of whom had biclonal gammopathies. The evidence that these two IgH-VDJs represent biclonal populations is unequivocal. For the 12 patients reported here, molecular analysis and single-cell PCR definitively show that second VDJs are productively rearranged IgH in independently distributing non-overlapping cell subsets. The MM IgH-VDJ and the second IgH-VDJ occur at different frequencies, in different anatomical locations, have different longitudinal patterns, in different cell types (PC vs B) and with different cytogenetics, identifying them as genuinely biclonal and excluding the possibility that they represent biallelic rearrangements. For those patients where single cell sorts were not available as a final definitive confirmation, the conclusion that they are distinct clones can still be reached by collective evidence from semi-qPCR, qPCR, relative peak heights of the two CDR3s in DNA fragment analysis, different chromosomal characteristics and changes of both relative CDR3 peak profile and clonal frequencies in the longitudinal analysis.

To date, we have not observed more than one partner clone. It may be that multiple clones do arise but at lower incidence rendering them undetectable. CDR3 analysis by DNA fingerprinting detects only dominant peaks that rise above polyclonal background. This also means that CDR3 analysis is unlikely to identify clonal IgM of the MM sequence [4], [16], [27] as this requires more sensitive detection using a clone-specific primer set.

The distribution of IgH-VDJ segment usage, mutational status and CDR3 length of molecularly-defined second clones are similar to those of MM clones. In only 3 cases were distinct differences noted: the use of VH4-34 gene in one clone and the use of unmutated VH genes with long CDR3s in two other clones. Intriguingly, some second clones share features common to other lymphoproliferative disorders. For example, in PT5, the second clone utilized VH1-69/DH3-3/JH6 with a long CDR3 (78 nt), similar to unmutated B-CLL [28]. In PT11 and PT12, intraclonal heterogeneity and multiple clonal isotype expression are similar to the pattern in Burkitt's lymphoma, follicular and diffuse lymphoma [29]–[31]. However, the clinical significance of second clones is not known given that none of these patients developed a clinical phenotype with features of these other B-cell disorders.

Longitudinal analysis characterizes the persistence of partner clones and their anatomical distribution. In PT6, IgG-κ and IgG-λ clones carrying different IgH VDJs resided in two different anatomical sites, one dominant in a sternal biopsy and the other dominant in serial iliac biopsies. Longitudinal analysis of PT3, clinically having only one M-protein, revealed an extremely high frequency for both partner clones that persisted over time and treatment. Although present at a relatively high frequency, the second clone in PT3 would not be considered leukemic [32]. Longitudinal analysis of PT5, whose molecularly-defined second clone predominated in blood, showed reduced clonal frequencies after treatment but molecular analysis indicated that second clone was not fully eliminated. Overall, our longitudinal studies showed that second clones can persist through the disease course but their clinical relevance requires further study on a case-by-case basis.

Chromosomal characteristics of molecularly-defined second clones were successfully identified in some patients despite technical limitations. For PT10 and PT6, non-overlapping chromosomal abnormalities were clearly detected in the MM clone and in the partner clone. For PT3, chromosomal abnormalities were seen only in MM cells, not in the second clone. Immuno-FISH was performed for several other patients, but interference by undefined numbers of normal B-lineage cells compromised interpretation of the results. Nevertheless, we did detect chromosomal abnormalities in the subset of B-cells with the same isotype as a given second clone, suggesting that chromosomal abnormalities may characterize some members of these second clones.

In a recent study [33], secondary MGUS was reported in MM, especially for those who received stem cell transplantation (SCT). Molecularly-defined second clones described here are different from secondary MGUS since nearly all were identified at diagnosis. Moreover, none of our patients had SCT and for most, clinical data did not indicate the presence of a second M-protein.

Molecularly-defined second clones may be present in the repertoire several years before extensive clonal expansion occurs. For PT8, a second clone was readily detectable at the time of relapse, seven years after diagnosis. This patient had been treated with thalidomide for the first six years and responded well. Yet by using highly sensitive nested RT-PCR, the second clone was molecularly detected in PB at diagnosis and in year 1. This confirms that the second clone was actually present but at a low frequency and dormant for at least two years. Although it is impossible to confirm with certainty that treatment played a role in the expansion of this second clone, it seems possible that exposure to thalidomide may have compromised the normally strong dominance of the MM clone and ultimately allowed this second clone to expand to levels detectable by CDR3 analysis. Alternatively, the drug may have changed the marrow niche leading to a more clonally permissible setting in which the minor clone could flourish.

Although it has been reported that some second malignancies share a clonal origin with the primary B lineage cancer [22], patients with two malignancies are often shown to originate from two separate clones [34], [35]. In our studies, biclonal origins were also shown in two patients with biclonal gammopathies and a patient with concomitant MM and CLL. Frequent occurrence of second clones thus suggests that transformation events may have occurred more often than originally thought and progression to malignancy if it occurs, is a rare event that may quickly establish dominance. The relationship between the second clones reported here and their clinical impact remains to be determined. It is however worthy of further study, especially given that it is now common for patients to be treated in the 3^rd^ and 4^th^ line setting. Characterizing the evolutionary path of the clinically relevant clones over the course of disease will be extremely important in devising appropriate therapy. The data here demonstrates in addition to the more common intraclonal diversity a substantial number of cases also demonstrate interclonal diversity. Several groups have shown “tides” of clonal evolution as MM progresses [36]–[40]. These investigators conclude that the observed genetic changes occur within the MM clone itself although this has not yet been directly shown. The work reported here shows that in addition to evolution by the MM clone, there are secondary, genetically unrelated clones (defined by a separate VDJ sequence) that are identifiable within the marrow and peripheral blood. This serves as a caution that any potential genetic signals of clonal evolution attributed to the clinically relevant dominant MM clone must be confirmed as such. Given that up to 16% of patients possess a second B-cell clone there is a risk that some of these features may be present not in the clinically relevant MM clone but rather in the unrelated clonal population.

In conclusion, a subset of MM patients carries two clones: a clinically-related clone and a molecularly-identified second clone. Second clones may represent potential precursors of second B cell malignancies, expanded bystander clones or may arise as part of anti-myeloma immune responses, but this remains to be evaluated. Second clones in MM originate from distinct parent B-cells as shown by the unrelated IgH-VDJ sequences of clinically defined and second clones. This suggests that MM may be characterized by multiple transformation events, culminating in the emergence of a dominant primary clone that becomes clinically detectable. For those patients with two clones, the second may have escaped dominance by the first, or a more clonally permissible setting may arise. As shown here, second clones persist over time and are present in PB and/or BM at frequencies several logs higher than those of antigen-stimulated normal clones characterizing them as distinct entities. As they expand, they may become susceptible targets for other transformation events. The presence of chromosomal abnormalities in some second clones suggests that they result from events that may predispose them to malignant transformation, as does their extensive clonal expansion in some patients. Even though their clinical relevance is not yet known, the identification of second clones indicates that future studies will need to confirm the relatedness of important myeloma clones and sub-clones. Overall, the clonal “communities” in MM appear to be more complex than previously appreciated. The frequent detection of second clones adds a new dimension to evaluating related and unrelated clonal expansions in MM and the role of treatment in clonal dynamics.

## Supporting Information

Table S1 **Summary of primer sequences.**
(DOC)

Table S2 **Combinations of primer sets for two-stage PCR.**
(DOC)
