# Supplementary material for: Frequent Occurrence of Highly Expanded but Unrelated B-Cell Clones in Patients with Multiple Myeloma
Source: PLoS One. 2013 May 28;8(5):e64927. doi: 10.1371/journal.pone.0064927 (PMC3665682; doi:10.1371/journal.pone.0064927)
Supplement: Table S1 — Summary of primer sequences. (DOC) [file pone.0064927.s001.doc]

**Table S1.** Summary of primer sequences

| **Primer** | **Sequence** |
| --- | --- |
| FR1c | 5’GGTGCAGCTG(G/C)(A/T)G(G/C)AGTC(G/A/T)GG3’ |
| FR3 | 5’CCGAGGACACGGC(T/C)(C/G)TGTATTACTG3’ |
| JHc | 5’ACCTGAGGAGACGGTGACC(A/G)(G/T)(G/T)GT3’ |
| CμB | 5’GGAATTCTCACAGGAGAC3’ |
| CδB | 5’GTGTCTGCACCCTGATAT3’ |
| CγB | 5’GGGGAAGACCGATGGGCCCT3’ |
| CαB | 5’GAGGCTCAGCGGGAAGACCTT3’ |
| qPCR β_2_m 5’ | 5’TTGTTGGGAAGGTGGAAGCTCAT3’ |
| qPCR β_2_m 3’ | 5’AACCAGACACATAGCAATTCAGG3’ |
| β_2_m 5’ | 5’CCAGCAGAGAATGGAAAGTC3’ |
| β_2_m 3’ | 5’GATGCTGCTTACATGTCTCG3’ |
| β_2_m 5’int | 5’TGTCTTTCAGCAAGGACTGG3’ |
| β_2_m 3’-1 | 5’ATCAGATGGGATGGGACTCA3’ |
| β_2_m 3’-2 | 5’CTACTCATACACAACTTTCAGC3’ |
