# Supplementary material for: Frequent Occurrence of Highly Expanded but Unrelated B-Cell Clones in Patients with Multiple Myeloma
Source: PLoS One. 2013 May 28;8(5):e64927. doi: 10.1371/journal.pone.0064927 (PMC3665682; doi:10.1371/journal.pone.0064927)
Supplement: Table S2 — Combinations of primer sets for two-stage PCR. (DOC) [file pone.0064927.s002.doc]

**Table S2. Combinations of primer sets for two-stage PCR**

|  |  | **Primer set** | |
| --- | --- | --- | --- |
| **Analysis** | **Product** | **First PCR** | **Second PCR** |
| Single-cell PCR | Clonotypic VDJ or β_2_m | FR1c/JHc plus β_2_m 5’int/β_2_m 3’-1 | CDR1/CDR3 or CDR2/CDR3 or β_2_m 5’int/β_2_m 3’-2 |
| Bulk PCR or RT-PCR | Clonotypic VDJ | FR1c/JHc | CDR1/CDR3 or CDR2/CDR3 |
| Bulk PCR or RT-PCR | β_2_m | β_2_m 5’/β_2_m 3’ | β_2_m 5’int/ β_2_m 3’ |
